# Emerging and Novel Functions of Complement Protein C1q

**DOI:** 10.3389/fimmu.2015.00317

**Published:** 2015-06-29

**Authors:** Lubna Kouser, Shanmuga Priyaa Madhukaran, Abhishek Shastri, Anuvinder Saraon, Janez Ferluga, Maha Al-Mozaini, Uday Kishore

**Affiliations:** ^1^Centre for Infection, Immunity and Disease Mechanisms, College of Health and Life Sciences, Brunel University London, Uxbridge, UK; ^2^Centre for Biotechnology and Bioinformatics, Jawaharlal Nehru Institute for Advanced Studies, School of Life Sciences, Secunderabad, India; ^3^St. Ann’s Hospital, Dorset Healthcare University NHS Foundation Trust, Poole, UK; ^4^Department of Infection and Immunity, King Faisal Specialist Hospital and Research Centre, Riyadh, Saudi Arabia

**Keywords:** complement, C1q, apoptosis, neurogenesis, pregnancy, cancer

## Abstract

Complement protein C1q, the recognition molecule of the classical pathway, performs a diverse range of complement and non-complement functions. It can bind various ligands derived from self, non-self, and altered self and modulate the functions of immune and non-immune cells including dendritic cells and microglia. C1q involvement in the clearance of apoptotic cells and subsequent B cell tolerance is more established now. Recent evidence appears to suggest that C1q plays an important role in pregnancy where its deficiency and dysregulation can have adverse effects, leading to preeclampsia, missed abortion, miscarriage or spontaneous loss, and various infections. C1q is also produced locally in the central nervous system, and has a protective role against pathogens and possible inflammatory functions while interacting with aggregated proteins leading to neurodegenerative diseases. C1q role in synaptic pruning, and thus CNS development, its anti-cancer effects as an immune surveillance molecule, and possibly in aging are currently areas of extensive research.

## Introduction

**Figure 1:**
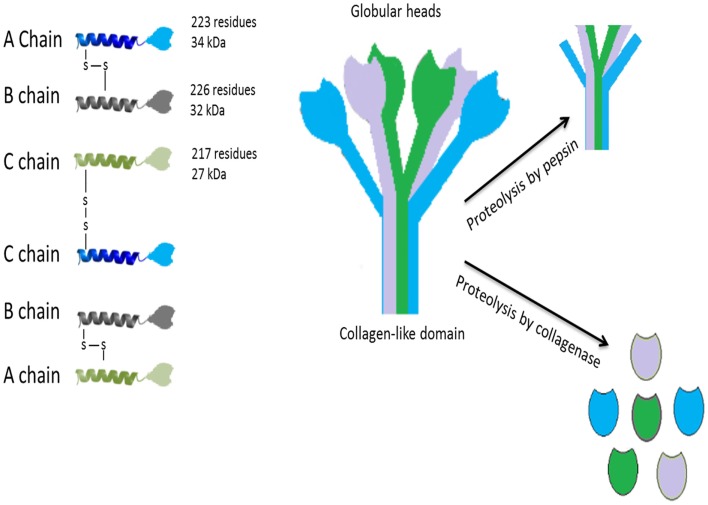
**Structure of C1q**. The hexameric C1q molecule (460 kDa) has a tulip-like structure, composed of nine non-covalently linked subunits of six A, B, and C chains; A–B dimer is 52,750 Da and C–C dimer is 47,600 Da. Fragments of C1q after pepsin digestion at pH 4.4 contain six intact connecting strands and small non-collagen-like peptides. After partial proteolysis with collagenase at pH 7.4, the globular regions (gC1q) appear intact. C1q binds to Fc regions of IgG or IgM, HIV-1, phosphatidylserine, HTLV-1, C-reactive protein, damaged neurons, myelin debris, amyloid fibrils, and apoptotic cells via the gC1q domain. The collagen-like region binds to serine proteases such as C1r and C1s leading to activation of the classical pathway. Collagen region of C1q also binds to C1q receptors (most notably, cC1qR or calreticulin using CD91 as a cell-surface adaptor) to augment phagocytosis by phagocytic cells and mount a pro-inflammatory immune response.

C1q, the first recognition subcomponent of the complement classical pathway, is a 460 kDa hexameric glycoprotein (1–3), which is composed of 18 polypeptide chains, composed of 9 non-covalently linked subunits having 6 of A (223 residues; 34 kDa), B (226 residues; 32 kDa), and C (217 residues; 27 kDa) chains (Figure 1). These chains have a short N-terminal region (3–9 residues), a collagen region having 81 residues and a C-terminal globular (gC1q) domain of about 185 residues (3, 4). The hexameric C1q molecule has a tulip-like structure made up of structural units, which combine in the fibril-like central portion (5). Crystal structure of the heterotrimeric gC1q domain, solved at 1.9 Å resolution, revealed an almost spherical (diameter of 50 Å), dense hetrotrimer associated by non-polar forces with Ca^2+^ ion bound at the top, showing a classical jellyroll topology (6). A strong structural homology with gC1q domain [mouse ACRP30, human collagen X, mouse collagen VIII (α1) and human C1q] has given rise to a C1q family with a β sandwich jellyroll topology similar to tumor necrosis factor (TNF), and hence, a C1q–TNF superfamily (7, 8).

C1q is a pattern recognition molecule as it has the ability to identify various structures and ligands on microbial surfaces, apoptotic cells, or indirectly via antibodies and C-reactive protein (CRP). C1q can bind IgG, IgM, HIV-1, phosphatidylserine (PS), HTLV-1, CRP, and many other ligands via gC1q domain, and also to various receptors including calreticulin-CD91, and integrin α_2_β_1_ (4, 9). Binding of C1q to most of its ligands is via gC1q domain, which involves recognition of charged patterns/clusters (7). Recently, a number of functions of C1q have been described that do not involve classical pathway activation, suggesting that C1q has an additional role to play in homeostasis and development.

## Modulation of Dendritic Cells Maturation and Functions by C1q

Dendritic cells (DCs) are antigen capturing and presenting cells of innate immunity (10, 11). Interstitial/dermal DCs (IDDCs) produce significantly more C1q at 48 h, following stimulation with IL-4 and granulocyte macrophage-colony stimulating factor (GM-CSF), as compared to Langerhans cells (12, 13). C1q has been shown to be produced by renal DCs in a mouse model of progressive lupus nephritis. Renal sections of mice showed significantly higher expression of C1q in tubulo-interstitium of old MRL/lpr mice as compared to young MRL/lpr mice. Increased co-localization of MHC-II^+^ cells with C1q was detected in old MRL/lpr mice (5 months) with lupus nephritis. Production of C1q derived from bone marrow precursors showed expression of C1q similar to other local sources such as macrophages. Furthermore, human kidney biopsies of systemic lupus erythematosus (SLE) patients showed an increased production of C1q by renal DCs (10).

C1q binds monocyte-derived immature DCs causing the NF-κB nuclear translocation from the cytoplasm. C1q enhances maturation of DC exhibiting raised surface expression of CD83, CD86, HLA-DR, and CCR7. Both gC1q and collagen region can independently induce DC maturation. In the presence of C1q, DCs secrete more IL-12p70 than immature DCs (14). C1q-primed mature DCs induce production of IFN-γ by co-cultured T lymphocytes (Th1 cells), stimulated by IL-12, and secreted by C1q-treated mature DCs (14). C1q produced by DCs is capable of activating complement and binding to apoptotic cells. C1q production by DCs is suppressed by IFN-α (15). Interestingly, C1q inhibits IFN-α production by plasmocytoid DCs (pDCs) induced by immune complexes (ICs), but enhances IFN-α production induced by herpes simplex virus (16).

Leukocyte-associated Ig-like receptor (LAIR; a transmembrane protein and a member of immunoglobulin super family) (17) is expressed on NK cells, B cells, monocytes, human fetal thymocytes, and T cells. LAIR 1 is an inhibitory receptor, which is considered to be involved in down-regulation of immune response (17). C1q binding to LAIR leads to the activation of LAIR-1 signaling. C1q inhibits the GM-CSF and IL-4 stimulated differentiation of monocytes into DCs. Interaction of C1q with LAIR-2 reduces the expression of LAIR-1 and CD14, implying that the C1q-mediated differentiation of DCs is reversed by LAIR-2 (18). C1q and LAIR-1 also function to inhibit IFN-α production by plasmacytoid DCs of either healthy or SLE individuals. This suggests that C1q and LAIR-1 interaction is involved in the arrest of monocyte differentiating to DCs, and in the inhibition of plasmacytoid DC activation (18). Furthermore, C1q expression by DCs can be localized at the arterial wall. Atherosclerotic lesions showed intracellular and extracellular expression of C1q, particularly the necrotic core surroundings were C1q-positive. C1q expression was not only limited to DCs but also to other cell types such as macrophages, neovascular endothelial cells within atherosclerotic plaques, endothelium of vasa vasorum in the adventitia, and in very few luminal endothelial cells. The expression of DCs in the arterial lesions was heterogeneous, implying that some DCs may reside in the arterial wall for local synthesis of C1q (19). Consistent with this, C1q binding to apoptotic cells enhances the phagocytosis and stimulates the production of IL-6, IL-10, and TNF-α by DCs, without affecting IL-12p70 production.

C1q binds to resting as well as activated B cells. C1q may be involved in the stimulation of B cells to either produce IgM or IgG isotypes in normal individuals or those with impaired B cell activity (20). Studies on C1q deficient^(−/−)^ mice have demonstrated that positive selection of B cells and IgM autoantibodies is increased by intracellular self-antigens that are displayed by dying cells, and the negative selection of B cells is decreased. The positive selection by self-antigens may be involved in the IgM and C1q-dependent clearance of dying cells, limiting the contact of conventional B cells to immunogenic self-antigens (21). C1q deficient mice developed splenic hypercellularity, displaying signs of accelerated CD4^+^ T cell activation, enhanced splenic plasma cells and total serum IgM levels. This suggests splenic monocytosis is caused by C1q deficiency as well as the activation of T cells in an autoimmune-prone mouse strain (22). C1q deficient mice also develop lupus-like disease and the apoptotic cells have elevated levels of autoantigens as these are not cleared by complement. Mice genetically deficient in C1q displayed high levels of IgM and IgG3 (23).

## Clearance of Apoptotic Cells by C1q and Its Role in Immune Tolerance

### Waste disposal and self-tolerance hypotheses

Apoptosis plays an integral part in development and homeostasis, through tissue and organ remodeling and in homeostatic cell turnover (24–26). C1q is important in development, and has a major role in clearing apoptotic cells, which may otherwise encourage autoimmunity. C1q deficiency predisposes one with a high risk to develop SLE, a prototype of autoimmune inflammatory disease (27). Two main hypotheses on C1q related autoimmunity have been proposed, being apparently complementary. In the first “waste disposal hypothesis,” due to C1q deficiency in clearing apoptotic bodies, an accumulated bulk of altered self apoptotic neo-antigens may induce in lymphatic tissues an uncontrolled affinity maturation and IgG isotype switching of specific self-reactive B cell clones in a T cell dependent manner (27). According to the second “self-tolerance hypothesis,” C1q and C4 also maintain peripheral tolerance-anergy of naturally occurring IgM producing self-reactive B cell clones against specific apoptotic antigens such as histone and dsDNA as part of apoptotic cell homeostasis. Some of these clones may be eliminated through their negative selection centrally in bone marrow and spleen (28). Nuclear antigens are highly conserved and may cross-react with bacteria. Their autoimmunity is typical of SLE (29, 30).

Apoptosis is an evolutionarily preserved death program mediated by caspase proteases and regulated by pro-apoptotic and anti-apoptotic signals of adjacent cells (24). Affected cells undergo characteristic cell shrinkage, non-random nuclear protein and DNA fragmentation, plasma membrane blebbing, and exposure of PS on their membrane surface. Blebs-apoptotic bodies are rich in nuclear material including dsDNA targeted by autoantibodies in SLE (24). PS is an important early apoptotic molecule recognized by C1q (in addition to other target ligands as shown in Table 1) and by other phagocytic cell receptors, such as CD36 and TIM-4. It enables a rapid cell phagocytosis before their release of inflammatory molecules (31, 32).

**Table 1:** **Emerging non-complement functions of C1q**.

C1q functions	Mechanisms	Ligands/receptors	Reference
Canonical Wnt signaling, induces aging	C1s-dependent cleavage of the ectodomain of LRP6	Frizzled receptors (Fz)	(33)
		LRP5/6	
Invasion of epithelial and endothelial cells	C1q collagen region with the host cell-surface receptor interaction	*S. pneumoniae* serotype 35A (NCTC10319), *S. pneumoniae* strain D39 (serotype 2)	(34)
Clearance of bacterial pathogens	Gram-positive (*S. pyogenes*) and Gram-negative (*E. coli*)	Collagen region; outer membrane protein from Gram −ve bacteria	(34–36)
		Lipopolysaccaride, fibronectin, fibrin, fibrinogen	
Escape mechanism for *Streptococcus pneumoniae*	C1q and PepO interaction	Pneumococcal endopeptidase O (PepO)	(37)
Induction of apoptosis in cells overexpressing WOX1	Phosphorylation of N-terminal WW domain of WOX1	WOX1	(38)
Induction of angiogenesis	Vessel formation during wound healing	gC1q domain	(39)
Clearance of apoptotic cells	gC1q domain	PS	(40–44)
		dsDNA	
		Calreticulin	
		Annexin A2 and A5	
Tolerance induction	Immature DCs	C1q/gC1qR	(45, 46)
		cC1qR	
Developmental synaptic pruning	Microglia		(47)
Uptake and transmission of PrP^Sc^ from the gut to the brain	Conventional DCs	Calreticulin	(49, 50)
Forms complexes of PrP oligomers		gC1q domain	(51)
Development of DC	DC	gC1qR	(52)
Induction of tolerance phenotype		cC1qR	(53)
Tolerogenic effect (TGF-β1 release)	
Negatively influences BCR signaling; enhances tolerance	B cell		(54)
Induces anti-inflammatory response	B cells		(55)
Inhibits activation and proliferation	T cell	gC1qR	(54)

SLE is strongly associated with C1q and C4 deficiency. Hereditary C1q deficiency, which is very rare, confers in homozygous persons at an early age, a more than 90% risk of developing the disease (27, 56). Deficiency in C4 carries a disease risk of around 80%. However, SLE is a heterogeneous complex disease due to acquired C1q deficiency, although there may be remissions (57). Symptoms, which are very variable, include skin rash, light sensitivity, glomerulonephritis, and arterial-venous thrombosis. SLE is mediated by autoantibodies to various self-antigens, predominantly to C1q and apoptotic blebs containing nuclear antigens. Its hallmark is deposition of ICs with such antigens (58). Immunological changes can precede the disease, sometimes several years in advance (28).

C1q, C4, and C3 proteins are involved in specific antigen presentation by DCs to T and B cells. DCs are central in processing self and non-self-antigens during immune response (59). In a physiological steady state, tissue immature migrating DCs sample microbe and tissue antigens, and in draining lymph nodes differentiate into follicular DCs, where they mature and present processed specific antigens to naive T cells in a MHC-dependent manner. Such antigen specific Th cells, in turn, interact via their receptor with antigen specific B cell, which may require T cell help. FDCs can also directly present antigens to specific immature B cells in lymphoid cell compartment (29, 60). However, in most cases, for efficient antigen B cell receptor (BCR) activation, self or microbial antigens are covalently tagged with adjuvants C3d or C3dg fragments as opsonins (61).

### Possible involvement of C1q receptors in tolerance

Of a number of candidate C1q receptors, two receptors for C1q, cC1qR and gC1qR, have been largely documented in the literature. cC1qR (now known as calreticulin) was identified on phagocytic cell surface as a receptor for the collagen region of C1q (and collectins) (62). Another C1q receptor specific to the globular head region, gC1qR, has also been described (63). Like C1q, the two receptors also seem to have multiple specificity and functions. C1q receptors are C1q collagen-like domain binding calreticulin (cC1qR), and C1q globular domain binding protein receptor (gC1qR) p33 (63). Calreticulin is part of multi-protein-binding scavenger receptor complex CD91, of which β chain is a transmembrane signaling protein. C1q/cC1qR/CD91 complex bound apoptotic cells can be taken up by immature DCs through their micropinocytosis (42, 43). Curiously, CD91 can also directly bind C1q (65). gC1qR is also an ubiquitous co-receptor with multi-ligand interaction and effector properties in various tissues. For example, in immature DCs, it interacts with transmembrane DC-SIGN-1 lectin receptor (66).

C1q, through two of its receptors (cC1qR and gC1qR), is involved differentially in monocyte–DC development (52). C1q may thus be involved in modulation of a specific antigen-associated phenotype of DCs, such as tolerance/anergy in the case of apoptotic nuclear antigens (63). Upon these antigen stimulations, mature DC subsets express immunosuppressive cytokines dominated by TGF-β and IL-10 (67), which may impact on specific self-reactive B cell clone development (30). Moreover, tissue migrating DC subsets, in contrast to resident follicular DC derived from blood, have the capacity to transform CD4^+^ Th cells into peripheral CD4^+^ CD25^+^ FoxP3 T-regulatory cells (Tregs), specific for an antigen. For example, myelin specific Tregs strongly ameliorated experimental autoimmune encephalomyelitis (EAE) (68). A subpopulation of monocytes migrating from various tissues to lymph nodes, express only minimally DC markers, while ingesting antigens (69). Such DC apoptotic and other antigen specific tolerogenic bias are apparently impaired in C1q deficiency, which may result in autoimmunity.

C1q, as a molecular pattern sensor, can directly bind PS as well as to nuclear antigens including dsDNA exposed on apoptotic blebs/bodies of various tissue cells such as keratinocytes and vascular endothelial cells. These complexes may be rapidly taken up through its C1q receptors by non- and professional phagocytes such as immature DCs and macrophages (40, 70). This feature has been studied with UV-B irradiated early apoptotic HeLa cells. C1q strongly interacted via multiple regions of its gC1q domain with PS on apoptotic cells (41). Calreticulin binds to gC1q, as well as to C1q collagen-like domain (cC1q) (71, 72). gC1q domain also possesses a lectin-like region in recognizing late apoptotic bodies DNA, i.e., its deoxy-d-ribose form (73).

cC1qR and gC1qR contribute to the development of DCs, during which they become differentially expressed, and consequently to naïve T-cell antigen presentation and to antigen specific T cell–B cell interaction (52). Normally, a steady state relation between both C1q receptors may be maintained, which may be perturbed in C1q deficiency. DCs are major extrahepatic producers of C1 complex, which can become expressed on their surface, acting in a paracrine way. This may include facilitating antigen presentation to naïve T cells (74). Such anchored C1q might on contact with apoptotic cell, help to induce in DCs a tolerogenic phenotype. As studied with macrophages, an initial close contact with apoptotic cells, possibly by tethering receptors, is needed for these tolerogenic effects, including TGF-β1 release. This cell state appears to be independent of receptors, which mediate PS exposed apoptotic cell phagocytosis such as CD36 and α_v_β_5_ integrin, as tested with mice deficient in these genes (53).

### TIM and tolerogenic phenotypes

A PS-specific apoptotic cell phagocyte receptor, TIM4 (T-cell immunoglobulin- and mucin-domain-containing molecule) has been cloned from mice peritoneal macrophages (31, 32). Instead of apoptotic cells, PS coated nuclei ejected from erythroid precursors were used in hemophagocytosis tests. A recent study found TIM4 requirement for β1 integrin interaction for apoptotic cell engulfment (75). A striking property of apoptotic cells is an imposing induction in immune cells such as monocytes–macrophages and certain DC subsets of a tolerogenic–anergic cytokine phenotype including TGF-β1, IL-10, and PGE_2_ (42, 67, 71, 76). However, for DC maturation and antigen presentation, a preceding pro-inflammatory phase, possibly to apoptotic antigens, is required. TIM3 ligand galectin-9 was shown to mediate human DC maturation by inducing IL-12 and TNF-α cytokines through NF-κB activation, in synergizing with TLRs (77). By contrast, in Th cells, TIM-3 can have an inhibitory effect on Th1 inflammatory phenotype, as well as on macrophages where it down-regulates IL-12 production. TIM-3 ameliorates murine EAE (78, 79). In inflammatory conditions, such as viral infection, monocyte-derived DCs assume an IL-10 suppressive phenotype through PS dependent hemophagocytosis to relief inflammation (80). Recently, a key Treg subset has been described in mice, expressing C-type lectin receptor CD69 and FoxP3^+^CD69^+^, regulated by STAT5 and ERK pathway (81). CD69 is involved in maintenance of tolerance, in regulation of pro- and anti-inflammatory Th17–Th2 cell phenotypes, and in expression of TGF-β1. Patients with an active SLE were found to be deficient in CD^4+^CD25^high^ Treg cell suppressive function (82).

Besides the effects on DCs, C1q was previously shown to directly inhibit T cell activation and proliferation via its gC1qR expressed on these cells (54). This receptor is also expressed on B-cells, where it may have tolerogenic effect (83). It has been suggested that this C1q/gC1qR negative regulatory pathway may be involved in maintenance of peripheral self-tolerance to nuclear antigens (46, 63). TIM-3 takes part in apoptotic cell recognition and uptake by DC, in antigen cross-presentation to T cells, in amelioration of autoimmune disease, and in immune cell tolerance induction. TIM-3, together with TIM-4, suppressed autoantibody production (79, 84). TIM-3 negative effects on Th1 appear to be similar to that of C1q/gC1qR showing anergy of T cells (54). TIM-3 is also associated, gC1qR dependently, with regulation of suppressor of cytokine signaling-1 protein (SOCS-1) and of opposing STAT-1 (83, 85).

Clearance by C1q of apoptotic bodies is regarded to be an important protective function, since its impairment is associated with renal, vascular, and other tissue deposition of complement components and of apoptotic bodies. If not rapidly removed, the altered self-debris may become immunogenic, exposing cryptic epitopes, leading to induction of autoantibody production (27, 56, 86, 87). Paradoxically, SLE, despite C1q deficiency, is a complement activation inflammatory disorder. Perhaps, MBL-associated serine proteases (MASPs) coupled with classical pathway may substitute for C1q inflammatory function (43). Alternative pathway may also become involved (86). C1q would thus appear protective against the disease through its putative tolerogenic effects (30, 46). As shown with macrophages, apoptotic cell clearance receptors are separate from the ones mediating tolerance (53). Further supporting this view is the finding in mice deficient in mannose binding lectin (MBL), and in CD14 receptors. Such animals exhibited apoptotic cell and debris deposition in various organs, but did not develop a SLE-like autoimmune pathology (74, 88, 89).

According to self-tolerance hypothesis, C1q and C4 may through immature DCs and immature B cell responses maintain a threshold for negative selection of antigen specific self-reactive B cells (30). In SLE, the main immune targets are highly conserved ribonucleoproteins. Natural IgM producing B cells, specific against these proteins, are kept anergic by C1q, C4, and by Tregs. Inert B cells may escape their apoptosis when unduly activated in the absence of C1q or C4 (29, 30). Using a mouse model of SLE associated with C4 deficiency, it has been revealed that tolerance and anergy of self-reactive B cell clones are impaired, which is normally maintained through interaction with bone marrow myeloid cells, and by spleen negative clonal selection. In the absence of C4, more of such antigen specific B cells reach maturity in peripheral lymphoid compartment, effects furthered by accumulated nuclear debris (90). Perhaps, by appearing so influential in self-tolerance, C1q and C4 might function in epistasis, i.e., being interdependent in their cell signaling.

Self-tolerogenic C1q/gC1qR pathway could potentially endow C1q with a protective function, distinct from that of C1q in apoptotic debris removal (30, 46, 63). These two C1q properties in immature DCs seem to vary in their receptors they may engage, i.e., C1q/gC1qR and C1q/cC1qR, respectively (42, 46, 64). In addition to self-reactive B cell clone elimination, their peripheral suppression by Tregs may be diminished as seen in patients with diseases such as SLE and anti-phospholipid syndrome (APS) (82, 91).

In a physiological steady state, immature DCs of various tissues sample apoptotic cell and other debris in order to migrate to lymphatic tissue where they, as follicular DC, may undergo maturation and immune activation by either presenting specific antigens via MHC class II to naïve T cells, or be tolerized (59). A role of C1q in self-tolerance, as well as in apoptotic cell removal, would be consistent with murine SLE model, rendered deficient in their C1q A-chain gene as homozygotes (*C1q*^−/−^). Such mice had shortened life span, a quarter developing glomerulonephritis with apoptotic body deposits, and ICs with autoantibodies (92).

### C1q, autoimmunity, and B cell tolerance

B cells are major effectors in SLE autoimmunity. Fate of immature B cell stages to maturity is selected via their antigen IgM/IgG membrane receptor (BCR) signaling, and modulated by several positive and opposing co-factors receptor, including T cell help (60, 93, 94). In these events, membrane bound complement receptor 2 (CR2, CD21), as part of BCR complex, include the signaling co-receptor CD19. CD21 captures C3d/microbe or altered self-antigen complexes, for antigenic-specific BCR stimulation. At the same time, C3d/antigen ligated CD21 activates CD19, which amplifies antigen specific BCR signaling, synergistically with BCR (95). Antigen-sensitivity of BCR complex is further boosted by co-receptor CD21–C3d/Ag complex, which cross-links a number of BCR complexes on a B cell membrane by being polyvalent, e.g., in ratio 3-C3d/1-Ag_._ Such structures can lower an antigen activation threshold for its BCR, 100- to 10,000-fold (61). C3d/Ag complexes are generated on unprotected body cell or microbe surface antigens, to which C3b-thiol ester domain C3d (TED) makes covalent bonds with Ag-hydroxyl groups. These are captured, processed, and presented in draining lymph nodes to B cells by FDCs. CD19 co-receptor also mediates DC retention in germinal centers and their survival (96). CD19/CD21/BCR complex can be translocated to B cell lipid rafts, which can interact with other signaling receptors (97).

CD19 glycoprotein is involved in intrinsic Src-family kinase signaling, associated with Inositol 1,4,5-trisphosphate 3-kinase (IP3 3-kinase/IP(3)K), Ca^2+^ influx, and mitogen activated protein (MAP) kinase (97, 98). A further BCR co-receptor is Bruton tyrosine kinase (Btk), expressed constitutively, and positively modulating BCR signaling. As tested in CD19 – promoter – Btk transgenic mice, an over expression of Btk within their B cells via NF-κB elicited in such animals a SLE-like anti-dsDNA autoimmunity, showing expansion of specific plasma cells. This phenotype was due to impairment of antigen specific BCR negative selection (99). Btk gene deletion, on the other hand, resulted in programed death 1 (PD1) expression, B cell apoptosis, and in hereditary disease such as X-linked agammaglobulinemia. Normally, Btk may thus directly maintain a threshold on antigen specific B cell negative selection, although its expression modulation itself may be subject to other BCR co-regulators. B cell malignancy such as chronic lymphocytic leukemia (CLL) is associated with a dysregulated Btk, and ameliorated by its inhibitors (99).

CD19-co-receptor is closely associated with CD22, an inhibitory co-receptor of BCR signaling, a member of Siglec family (sialic acid-binding Ig-like lectin). On IgMmBCR ligation and via CD19 Lyn kinase, CD22 is activated through phosphorylation of its immunoreceptor tyrosine based inhibition motifs (ITIMs). These activated motifs then recruit inhibitory tyrosine phosphatases such as SHS-1, SHS-2, and inositol-phosphatase (SHIP), dephosphorylating their substrates, including CD19 cytoplasmic domain tyrosine residues, as a negative feed-back loop (100, 101). CD22 and Siglec-G defect and polymorphism in acetylesterase are associated with susceptibility to autoimmune disease (102, 103).

C1q, which is highly expressed on B cells, may negatively influence BCR signaling, and promote their tolerogenic capacity. It may be part of the negative C1q/gC1qR pathway, which suppresses CD4^+^ T cell activation and proliferation (54). Earlier studies with B cell lines, such Raji, Daudi, U937, and Molt4, demonstrated a C1q-induced anti-proliferative response (55). C1q promoted human peripheral blood and tonsillar B cell differentiation, stimulated with *S. aureus* Cowan (SAC), but did not affect their proliferation. C1q specifically induced IgG production over that of IgM, via its collagen domain, bound to B cell surface (20). Apparently both C1q receptors may be engaged in B cells with separate functions, depending on B cell stage and stimulation (20).

In a comparative study on C1q and on HCV core protein effects, via gC1qR ligation, C1q likewise did not enhance normal human CD20^+^ B cell proliferation, stimulated by phytohaemagglutinin (PHA). However, C1q, like HCV core protein, augmented CD69 expression, which is an activation marker and an immune suppressor (81, 83). By contrast, HCV core protein augmented B cell proliferation, co-stimulatory molecule expression, down-regulated suppressor of cytokine signaling-1 (SOCS-1), and up-regulated STAT-1 activation-phosphorylation. In T cell, it had opposite effects. These responses were gC1qR dependent, and associated with TIM-3 function. Such cell signaling dysregulation is thought to sustain the viral disease chronicity and autoimmunity (83, 85). C1q/gC1qR effects on T and B cells may be compatible with C1q being a tolerogenic protein against autoimmunity as in SLE (63). Its stimulatory effect on B cell CD69 expression may be relevant. CD69 is a type 2 lectin receptor, expressed on all bone marrow derived leukocytes. Its co-expression on a Foxp3 Treg subset facilitates its tolerogenic property. Potentially, it may also augment such effects of CD11b/CD22 receptors in B cell tolerance (104). CD69 is a negative regulator of leukocyte migration-egress of lymphocytes from lymphoid organs, inhibiting sphingosine 1-phosphate receptor-1 (S1P_1_) (81, 105). gC1qR is a multi-ligand binding adaptor protein to various cell receptors (45). In adipocytes, it serves as a positive co-receptor to insulin receptor tyrosine kinase in insulin signaling (106). In B cells, it might be a tolerogenic co-receptor.

### Autoantibodies against C1q

Autoantibodies against C1q collagen region have been strongly correlated with lupus nephritis, suggesting their pathogenicity (107). In a mouse model, it was revealed that anti-C1q autoantibodies were glomeruli damaging only in conjunction with complement fixing ICs with anti-nuclear protein and dsDNA antibodies deposits (108). Apparently, C1q also exhibits hidden epitopes when bound to various cell debris (107, 109). Anti-gC1q autoantibodies were detected in several SLE nephritis patients, which inhibited C1q binding to IgG and C-reactive protein. It has been suggested that such autoantibodies may represent an acquired C1q deficiency (110, 111). Secondary C1q deficiency is much more common in SLE and other diseases with variable frequency. However, only 20–50% of SLE patients have anti-C1q autoantibodies. Some disease free people also have anti-C1q autoantibodies, suggesting a need for an injury as a trigger. To sustain the disease, however, anti-nuclear antibodies and ICs, also mediated largely by C1q, are a hallmark for SLE (107).

Thus, C1q may distinctly contribute to apoptotic cell phagocytic clearance as well as tolerance maintenance to such natural self-antigens, effects which may be cooperative. C1q may employ in these tasks its cC1qR as well as its gC1qR in negative anti-inflammatory pathway of T and B cells. Both C1q receptor expression is required in DC differentiation and consequently in antigen presentation to T and B cells. On debris clearance, DCs may assume a tolerogenic, anergic state. Antigen stimulated T cells and B cells may respond similarly to C1q negative regulation. Autoimmune disease, such as SLE, is heterogeneous and complex. Its susceptibility may be increased not only by C1q defects but also by malfunction of other genes such as of BCR co-receptor Btk, CD22, TIM-3, and Tregs in BCR signaling. There is clearly a gene redundancy in apoptotic cell clearance mechanism. Thus, there is a good argument to explore the negative C1q/gC1qR pathway further in autoimmune disease.

## Role of C1q in Pregnancy

### Activation and regulation of complement in human pregnancy

Healthy normal pregnancy is associated with systemic activation of complement system (112–114). Placenta with the semi-allogenic fetal tissue is a potential target for complement-mediated immune attack (115, 116). During normal pregnancy, complement system is found activated by the presence of natural IgG in the normal placenta. This needs to be well regulated to control alloreactivity and placental inflammation (117). Complement products are found deposited on the placental tissues (118–121). Following decidual invasion of the fetal tissues, the fetus gets exposed to the complement system in the maternal blood at the intervillous space (122). In addition, extravillous trophoblasts (EVT), known as endovascular trophoblasts, migrate down the luminal walls of the spiral arteries and eventually enter the decidua and cause deep vascular changes in the spiral arteries producing apoptotic debri that favors complement activation with limited placental damage related to vascular remodeling process (123, 124). However, excessive or uncontrolled activation of the complement system can provoke adverse outcomes damaging the fetal tissues. Thus, for successful pregnancy, complement activation requires proper regulation.

**Figure 2:**
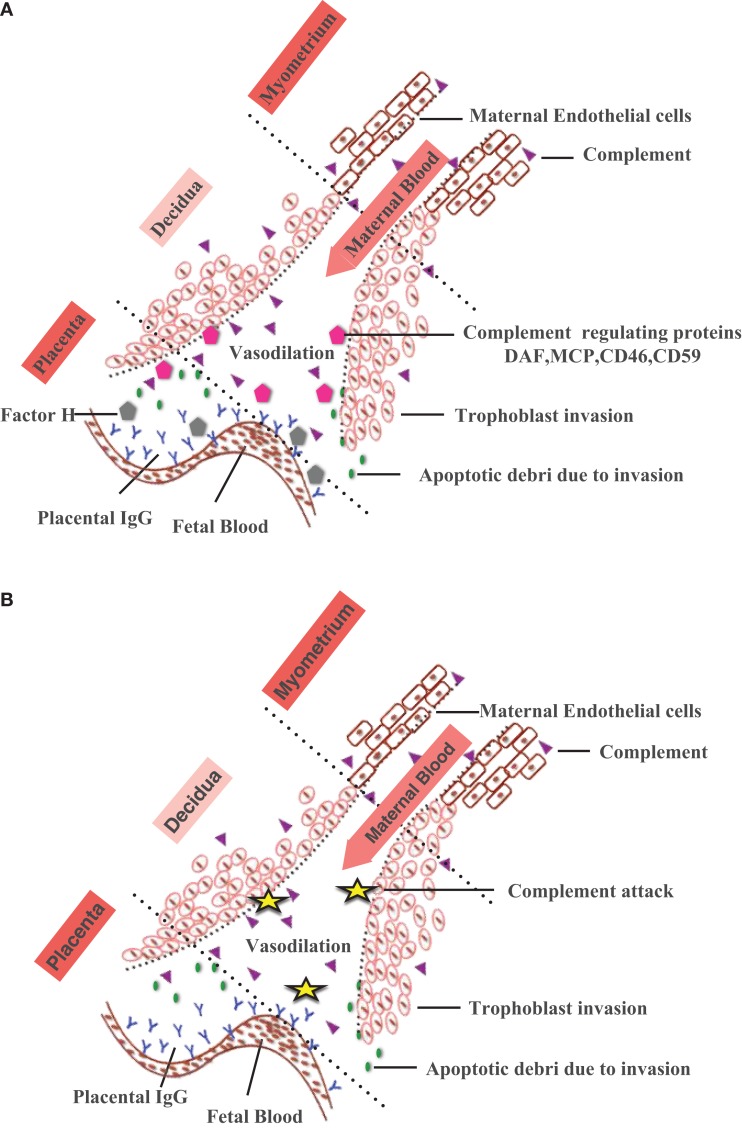
**Complement activation at the feto-maternal interface during pregnancy**. **(A)** During normal pregnancy, maternal endometrium transforms into feto-maternal interface (decidua) and surrounds the implanted embryo offering nutrition and protection against the maternal immune system. With advancing gestational age, decidua undergoes extensive tissue remodeling (trophoblast invasion and spiral artery formation) that results in apoptotic debris formation. The apoptotic debris and placental IgG activate classical pathway via C1q deposited at the feto-maternal interface. By eliminating the immune complexes and apoptotic cells, complement system protects the mother and fetus. Complement regulatory proteins found on human placental tissues such as Factor H, DAF, MCP, CD46, and CD59 prevent excessive complement activation. **(B)** In the absence of regulatory proteins (DAF, MCP, CD46, CD59), excessive complement activation results in improper placentation, characterized by increased ROS, inflammatory cytokines, angiogenic proteins (accumulation of apoptotic cells), improper vascularization, and spiral artery remodeling contributing to development of pathological/complicated pregnancies.

During normal pregnancy, fetus specific antibodies such as anti-HLA and anti-trophoblast antibodies in maternal serum bind C1q and activation of the classical pathway can have adverse pregnancy outcomes (125, 126). A number of complement regulators including factor H (117), decay-accelerating factor (DAF, CD55) (127), membrane co-factor protein (MCP, CD46), and CD59 (128, 129) are expressed locally on the surface of the cytotrophoblasts, syncytiotrophoblasts coating the villi, and EVT (130, 131). Thus, complement system at the feto-maternal interface defends the mother and the fetus against the invading pathogens; protects the fetus from the maternal immune system and maintains fetal tolerance (Figure 2).

### Distribution of C1q in the female reproductive tract

The levels of complement components gradually rise with gestation. C3, C4, C5, factor B, factor H, and factor I have been detected in amniotic fluid, umbilical cord blood (132–134), utero-placental spiral arteries (135), and placental tissue (136). Recently, deposition of early (C1q and C4) and late (C5, C6, C9) complement components has been reported in the placenta (123). Most notably, early components are detected in the stroma of the villi around fetal vessels, while late complement components are detected on the trophoblast membranes (118). Spiral arteries in the first trimester decidua show both early and late complement component deposits. C1q is produced by the trophoblasts and decidual endothelial cells (DECs) (118), human decidual stroma, and synthesized invasive EVT (118, 137). Recently, non-immune decidual cells such as invasive trophoblasts and stromal cells have been shown as a major source of C1q at the feto-maternal interface using first trimester human placenta (138).

### Role of C1q at the fetal–maternal interface during pregnancy

C1q is considered to have a key role in trophoblast invasion, spiral artery remodeling, and normal placentation (139). C1q is produced by DECs that connect the endovascular trophoblasts and the endothelial cells required for normal placentation such as trophoblast invasion into the decidua and vascular remodeling during pregnancy (118, 137, 140). In addition, it enhances trophoblast adhesion and migration by the activation of MAP kinase pathway via gC1qR/P33 integrin (137). Recently, transcriptional factor, PU.1 has been shown to be associated with the decidual C1q expression in trophoblasts and stromal cells. PU.1 transcriptional factor is associated with the development of hematopoietic myeloid lineage immune cells (141). Thus, the presence of PU.1 and C1q at the feto-maternal interface is likely to be involved in the regulation of trophoblast and stromal cell linage differentiation during early stages of pregnancy such as implantation and placentation (142).

### Association of C1q with disorders and complications of pregnancy

**Figure 3:**
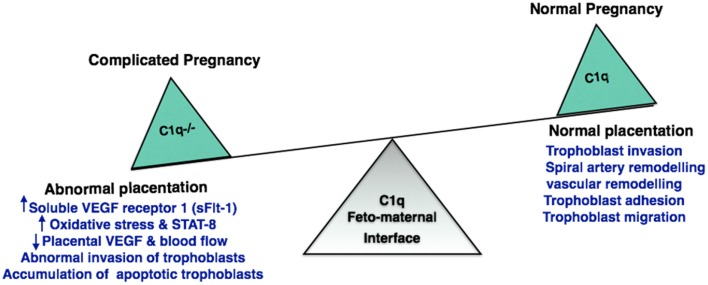
**Significance of C1q in normal and adverse pregnancy**. Absence of C1q results in abnormal invasion of fetal trophoblast into the decidua. C1q deficiency increases oxidative stress and accumulation of apoptotic trophoblasts. This has an adverse effect on placenta inhibiting the generation of placental vascular endothelial growth factor (VEGF) and blood flow, resulting in implantation failure and pregnancy complications such as recurrent pregnancy loss, miscarriage, abortion, and preeclampsia.

Excessive complement activation has been associated with adverse pregnancy outcomes such as miscarriages, preterm delivery, and preeclampsia. During pregnancy, EVT invades the decidua until it reaches the inner third of the myometrium. The extent of trophoblast invasion needs to be regulated. Inadequate tapering of maternal arteries and improper trophoblast invasion has been associated with preclampsia. It is a common disorder characterized by abnormal placentation. C1q^−/−^ mice show key features of preeclampsia, such as hypertension, albuminuria, endotheliosis, decreased placental vascular endothelial growth factor (VEGF), increased soluble VEGF receptor 1 (sFlt-1), increased oxidative stress, decreased blood flow, increased fetal death, diminished litter size, abnormal invasion of trophoblasts, and increased levels of STAT-8 (inhibitor of trophoblast migration) (139). Furthermore, C1q^−/−^ mice are unable to clear apoptotic trophoblasts whose accumulation can result in abnormal placentation (92). Differences in the C1q deposition in placental samples between early and late-onset of preeclampsia groups can act as a good biomarker for preeclampsia (143). It appears that C1q produced by trophoblasts are responsible for impaired trophoblast migration and abnormal placentation. It is also likely that the trophoblast C1q is required for the cross-talk between the EVT and DEC to promote trophoblast migration, vascular remodeling, and normal placentation process (140) (Figure 3).

Normal pregnancies are characterized by increased anti-C1q autoantibodies in the serum when compared to complicated pregnancies. Ectopic pregnancies have the lowest levels of anti-C1q antibodies with increased IL-15. Thus, IL-15 and anti-C1q antibodies are good serum biomarkers for missed abortions (144). Clinically, spontaneous miscarriage, still birth, and fetal resorption have been detected in mothers exposed to polychlorinated biphenyls (PCBs) (145). In patients with spontaneous abortion, PCBs content is associated with increased expression of gC1qR, and reduced percentage of apoptotic cells. Thus, gC1qR may be relevant for PCBs-mediated trophoblast cell apoptosis (146). Villitis of unknown etiology (VUE), characterized by inflammation in the chorionic villi, has been associated with miscarriage (147). In a study using placental samples with and without VUE, the distribution and amount of C1q, C3d, IgG, and IgM in the chorionic villi were examined. The only difference observed between two groups was the abnormal distribution of C1q in the stromal villi (148), suggesting involvement of C1q in villitis.

Several pathogens including *Trypanosoma cruzi*, cytomegalovirus, *Listeria monocytogenes*, and *Neisseria gonorrhea* can infect the decidua, and then spread to placenta and chorioamniotic membrane. When left untreated, the chorioamnionitis can lead to preterm birth, intrauterine growth retardation, still birth, and other complications of pregnancy (149).*T. cruzi* protozoan infection that spreads to humans by blood sucking bug *Triatominae* (150) can be transplacental, being passed from the mother to the fetus (151). When the mother is infected, the *T. cruzi* calreticulin (TcCRT; 45 kDa), migrates from the endoplasmic reticulum to the external surface of the parasite and binds to the maternal C1q (152). TcCRT bound maternal C1q recognizes/binds to CRT in the placenta. Thus, TcCRT bound maternal C1q acts as a molecular bridge to spread *T. cruzi* infection from the mother to the placenta. TcCRT, a potent virulence factor, enhances infectivity in the host by preventing the activation of the classical pathway and angiogenesis (153, 154). By enhancing the cross-talk between the CRT found on the surface of the parasite and its receptor on human placenta, maternal C1q is thought to play crucial role in spreading *T. cruzi* infection (155). During pregnancy, there is a possibility of increased disseminated gonococcal infection. Fetus remains at an increased risk due to neonatal sepsis, arthritis and systemic illness. Under *in vitro* conditions, C1q incubated with *N. gonorrhoeae* increases the gonococcal cell infection instead of clearing the gonococcal bacteremia (156). DECs have previously been shown to synthesize C1q during pregnancy (118). DECs, as opposed to endothelial cells isolated from adult skin, display decreased levels of TLR-4, MD2, and MyD88. This raises the possibility that C1q at the feto-maternal interface may contribute to immunoregulation of excessive inflammation caused by infection during pregnancy (157).

### Potential therapeutic agents to prevent C1q-associated pregnancy disorders

Heparin, a well-known anti-coagulant, has been shown to reduce pregnancy complications in women with anti-phospholipid (aPLs) antibodies (158, 159). It prevents apoptosis of human trophoblasts (160), enhances placental proliferation (161), and reduces EVT invasion (162). Heparin binds to the C1q collagen region following gC1q binding to ICs, thereby reducing the classical pathway activation (163, 164).

Animal studies have shown use of statins as a promising therapeutic strategy in preventing the onset of preeclampsia. Pravastatin treatment in C1q^−/−^ mice prevents the key features of preeclampsia by lowering blood pressure, and improving vascular reactivity, endothelial function, and normal placental VEGF. The study highlights the effectiveness of provastins in order to improve placentation and pregnancy outcomes (139).

## Role of C1q in the Pathophysiology of the Central Nervous System

**Figure 4:**
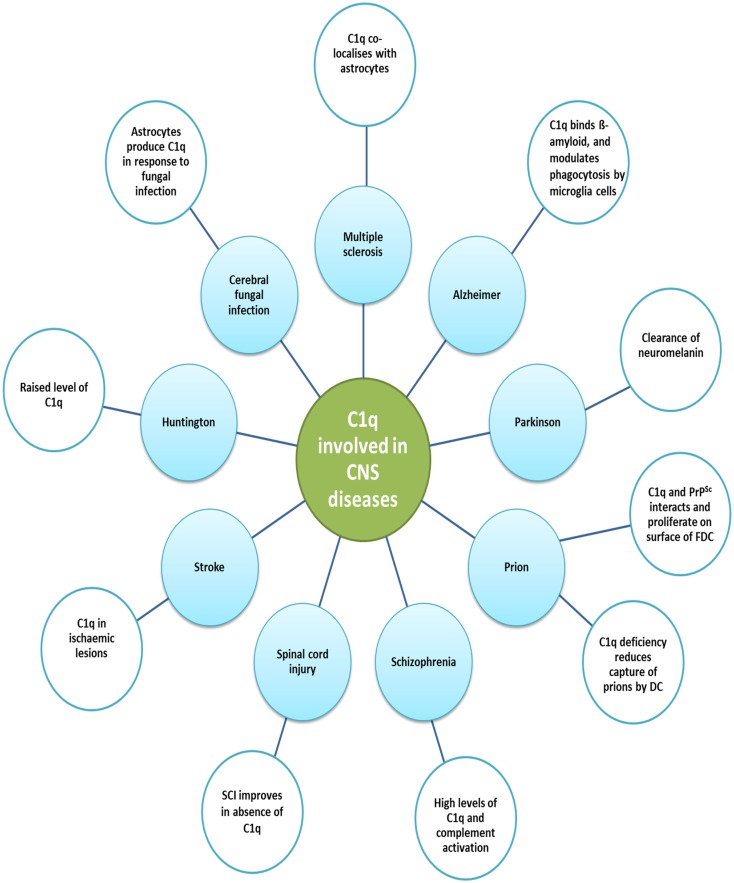
**Various diseases of central nervous system and the involvement of C1q**. various functions of C1q in the central nervous system and the associated diseases are illustrated. Increased levels of C1q have been found in Huntington disease and schizophrenia. In Huntington disease, striatal volume is decreased in mouse models; however, cell number does not differ. Prion disease is a result of undue loss of neurons and misfolded prion protein, PrP^Sc^, deposition. Early synaptic failure and neuronal loss affect behavioral symptoms, indicating the early onset of disease. C1q deficiency reduces the capture of prions by DCs, as it is only involved in the primary phase of the disease, transmitting prions to the CNS. C1q production is triggered in the CNS by astrocytes in response to cerebral fungal infection. The role C1q plays in Parkinson’s disease includes the opsonization of neuromelanin and phagocytosis by microglia cells. Complement pathway up-regulation in brain regions can be observed in AD, which may be due to amyloid β peptides binding within collagen-like domain of C1q, thus activating classical pathway. In addition, fibrillar amyloid β interaction with C1q via activates the classical pathway. Microglia cells, which express C1q in the substantia nigra pars compacta of Parkinson disease, phagocytose and clear debris of degenerating neurons.

Many components of immune system including complement proteins are locally produced in the CNS in order to maintain homeostasis. Similar to its role in the peripheral system, C1q is associated with protection of the CNS against infections such as bacterial meningitis. Low-level complement activation is also considered to be beneficial in the removal of toxic or aggregated proteins. However, sustained or chronic activation can be detrimental to the CNS due to microglial activation and production of pro-inflammatory cytokines. Therefore, neuroinflammation plays a role in the maintenance of homeostasis or neuroprotection as well as neurotoxicity, depending upon the level of complement activation (165–167). Apart from having an important role in CNS infections, traumatic brain injury, neurodegenerative diseases, and other neurological and psychiatric conditions (Table 2; Figure 4), a novel role for C1q has been established in the pathogenesis of prion diseases, development of CNS and synaptic pruning.

**Table 2:** **Role of C1q in CNS diseases**.

CNS disease	Role of C1q	Reference
Prion diseases	Initial uptake of PrP from gut; propagation and transmission to CNS	(166)
Alzheimer’s disease	C1q binds to β-amyloid via B chain of gC1q region and modulates phagocytosis by microglia	(2, 168)
	Absence of C1q causes less neuropathology in AD mouse models	(169)
	Co-localization of C1q with neurons is observed in pre-clinical stage of AD	(170)
	Increase in reactive oxygen species in neurons incubated with C1q leading to neurotoxicity	(171)
	In the presence of C1q, the neurotoxic effects of Aβ and SAP are reduced	(48)
Parkinson’s disease	Neuromelanin opsonized by C1q and phagocytosed by C1q-positive microglia	(172)
Multiple sclerosis	Co-localization of C1q with reactive astrocytes in CNS plaques	(173)
Schizophrenia	Increase in serum C1q and increased total complement activity in serum	(174)
Spinal cord injury (SCI)	Deficiency of C1q improves recovery following SCI	(175)
Stroke	Presence of C1q in ischemic lesions	(176)
	C1q deficiency found to be neuroprotective in hypoxic–ischemic brain injury	(177)
Huntington’s disease	Increased C1q co-localization in striatum	(178)

Microglia, astrocytes, and neurons are known to be good sources of complement proteins especially under stress, injury, ischemia, or infection. Microglia are the specialized macrophages in the CNS that constantly survey their surroundings. Upon being activated by environmental or external stimuli, microglia retract their ramifications and appear amoeboid. Production of C1q by microglia is at low level in the resting phase, which increases upon activation, leading to production of pro-inflammatory cytokines that can cause neuronal cell death (179). C1q binds to apoptotic cells and neuronal blebs thereby enhancing phagocytosis by microglia. Farber et al. (180) found that C1q stimulated microglial synthesis of TNF-α and IL-6, which is considered to disrupt blood–brain barrier. Moreover, C1q released by activated microglia maintained and balanced the level of activation of microglia in an auto- or paracrine manner. Interestingly, microglia stimulated with C1q *in vitro* were found to decrease LPS-induced production of TNF-α and IL-6 (181). Astrocytes have been shown to produce C1q in response to cerebral fungal infection (182). Astrogliosis refers to change in morphology and hypertrophy of astrocytes, which leads to scar formation. This occurs in response to CNS insult and is associated with C1q and cytokine secretion. Furthermore, co-localization of C1q with reactive astrocytes is a significant feature of CNS plaques seen in multiple sclerosis patients (173). Neurons also produce C1q *in vitro* (183). For example, hippocampal cells secrete C1q in response to β-amyloid (184). Presence of C1q protects neurons against β-amyloid and serum amyloid P-induced neurotoxicity (48). C1q-mediated neuroprotection involves up-regulation of genes associated with cholesterol and lipid distribution/metabolism such as cholesterol-25-hydroxylase and insulin induced gene 2. Addition of C1q leads to decreased levels of cholesterol in neurons, which is known to be a factor that causes outgrowth of neurites. C1q also directly affects nerve growth factor (NGF) by upregulating its transcription factors and downregulation of microRNA that target NGF (185). Thus, C1q (and the complement system) has a dual role: beneficial as well as detrimental to the CNS offering opportunities for therapeutic intervention in neuroinflammatory and neurodegenerative diseases.

### Prion diseases

Prion diseases or transmissible spongiform encephalopathies are a group of neurodegenerative diseases that are infective and are known to occur in humans, cattle, deer, and other livestock such as sheep and goat. Some of the diseases that affect humans include Creutzfeldt–Jakob disease (CJD), fatal familial insomnia, and kuru; while those involving animals include bovine spongiform encephalopathy and scrapie. Prion proteins are normally present in human body, although the fibrillar form of this prion protein (PrP^Sc^: scrapie-associated prion protein) are cytotoxic and form protease resistant fibrils and oligomers. PrP^Sc^ accumulates in neurodegenerative disease plaques. Histopathological features observed in prion disease affected brains include spongiform changes, gliosis, loss of neurons, accumulation of amyloid plaques, and synaptic degeneration in hippocampus (186–188).

C1q is involved in uptake, propagation, and transmission of PrP^Sc^ from the gut to the brain. With the exception of direct brain exposure due to neurosurgery, the mode of transmission for prions is through oral or parenteral route. C1q^−/−^ or C3^−/−^ mice have been found to have significantly delayed onset of disease after peripheral exposure to PrP^Sc^ (49). C1q plays a role in the initial uptake of PrP^Sc^ by conventional DC that express C1q receptor calreticulin (50). Absence of C1q has been found to significantly reduce the capture of prions by DC (189). Additionally, follicular DC is also considered to be a good source of endogenous monomeric prion protein. C1q interacts with PrP^Sc^ in an antibody-independent manner, which is considered to proliferate on the surface of follicular DC (190). Inactivation of follicular DC was found to delay neuroinvasion by PrP^Sc^ (191). *In vitro* studies using human microglial cells have also shown that the presence of C1q along with PrP causes an increase in fibril formation of PrP. This leads to an increased activation of microglial cells causing secretion of pro-inflammatory IL-6 and TNF-α (192). C1q has been found to enhance formation of PrP oligomers and form complexes with these PrP oligomers via its gC1q domain. This complex has the ability to activate classical pathway, and this is considered to prevent neuronal cell death caused by these oligomers of PrP (51). However, this interaction is considered to prevent the elimination of prion seed, thereby increasing its aggregation. Thus, C1q is involved in initial pathogenesis of prion disease by enhancing uptake of prions in the peripheral system, propagating into CNS, forming complexes with PrP oligomers, and increasing fibril formation.

## Synaptic Pruning

C1q (and the complement system) has been shown to play a vital role in synapse elimination during developmental stage of CNS as well as in synaptic pruning to increase efficiency of neuronal transmission. During developmental stage, a redundant growth of neural network occurs which also leads to excessive synaptic formations. This needs to be kept in check in order to maintain plasticity and ensure proper functioning of the CNS. Complement proteins, including C1q, have been localized at the sites of synaptic elimination in the mouse reticulogeniculate system, which is a model for studying synapse elimination during developmental stage (47). C1q is involved in synapse elimination during the developmental stage of CNS where postnatal neurons express C1q in response to an astrocyte-mediated signal and then co-localizes with developing synapses. C1q^−/−^ mice show failure of synapse elimination (47). Thus, role of C1q is considered to be akin to that in the peripheral system wherein C1q tags apoptotic cells. In the CNS, C1q is considered to tag immature or unwanted synapses leading to their elimination by microglia.

An important element of glaucoma includes death of retinal ganglionic cells (RGCs). In mouse retina, C1q is expressed only during the developmental phase. Interestingly, in glaucoma mouse model, C1q re-localizes to synapses in adult inner plexiform layer of the retina. This mechanism could be an important early event in glaucoma leading to C1q-mediated tagging of synapses, which, in turn, causes synapse loss and death of RGC. Furthermore, *C1qA* and *C1qB* chain gene expression was found to be markedly elevated (up to 25-fold) in mouse retinas with moderate glaucoma (47). More recently, TGF-β, secreted by astrocytes, has been shown to induce production of C1q by purified RGC (193). Disruption in cytokine signaling pathway of TGF-β leads to inhibition of synaptic pruning *in vivo* that is dependent on C1q and microglia. Furthermore, C1q has been also found to regulate microglia-mediated synaptic pruning in the thalamus (193).

Failure of synaptic pruning is an essential aspect of epileptogenesis. C1q^−/−^ mice show aberrant synaptic connectivity and spontaneous epileptiform activity is evident (194). This is likely due to the presence of excessive excitatory synapses. This failure of synaptic pruning also contributes to a significant increase in dendritic length, branching, and density of dendritic spines in C1q^−/−^ mice (195). In a rat model of temporal lobe epilepsy, an increase in C1q production by glial cell and neurons was observed in hippocampus following status epilepticus (196). Role of C1q in epilepsy is currently ambiguous. Due to an increased activation of complement, neuroinflammation can reduce threshold for seizures. On the flipside, its absence leads to spontaneous epileptiform activity due to deficient synaptic C1q function.

An increase in C1q level (up to 300-fold) has been observed in aging brain, especially in pyriform cortex, substantia nigra, and hippocampus (197). C1q immunoreactivity was detected in microglia throughout the early postnatal brain. Interestingly, C1q also co-localized with inhibitory (GABAergic) neurons in the hippocampal dentate gyrus. C1q was also abundant in close vicinity of synapses. Furthermore, the increase in C1q level in aging brain also correlated with cognitive decline. Indeed, synaptopathy or synaptic dysfunction is considered to be the one of the earliest feature of neurodegenerative diseases such as Alzheimer’s disease, Parkinson’s disease, Huntington’s disease, and Prion diseases (198, 199).

## Induction of Apoptosis in Cancer Cells by C1q

Human prostate cancer cells DU145 express WOX-1, which is known to be a tumor suppressor and pro-apoptotic protein. When DU145 cells cultured overnight in the presence of heat-inactivated serum (10%) followed by starvation for 1 h without serum were treated with exogenous C1q, it induced rapid accumulation of nucleic Tyr33-phosphorylated WOX-1 (p-WOX1) in comparison with the C1q-depleted serum treated cells where p-WOX1 was mainly localized in the cytoplasm (38). The C1q-treated DU145 cells adhered to the cover glass surface and a significant increase in the formation of clustered microvilli in between the cells was observed. Subsequently, the cell membrane shrunk, membrane blebbed, and eventually the cells underwent apoptosis. The inter-nucleosomal DNA fragmentation analysis showed the cleaved DNA ladders, which further established the C1q/WOX1-induced apoptosis in the DU145 cells.

The activation of WOX1 by C1q was further determined by culturing the EGFP–WOX1 and EGFP alone transfected DU145 cells overnight (in 10% heat-inactivated fetal bovine serum) followed by C1q treatment for 24 h. The C1q treatment resulted in activation of WOX1-induced apoptosis and growth suppression of the DU145 cells in the EGFP–WOX1 transfected cells whereas C1q did not induce apoptosis in the DU145 cells overexpressing EGFP vector only. These findings also suggest that WOX1 was a downstream effector of C1q-mediated apoptosis without the involvement of complement activation since the serum was heat inactivated.

Out of the two domains, N-terminal WW and C-terminal short chain alcohol dehydrogenase/reductase (SDR) of WOX1, the WW domain is believed to be involved in the C1q-induced activation of WOX1. This was shown by transfecting the DU145 cells with a dominant negative-WOX (dn-WOX1), which was designed with alterations in the N-terminal WW and is known to block the apoptotic function of p53 and prevent phosphorylation of endogenous WOX1 at Tyr33. The transfected cells resisted C1q-induced apoptosis when transiently overexpressed with dn-WOX1 (EGFP tag) whereas non-transfected cells underwent apoptosis. Furthermore, co-transfection of DU145 cells with N-terminal WW domain of WOX1 (WOX1ww) and dn-WOX1 showed a decrease in the C1q-induced apoptosis. These observations suggest that N-terminal WW domain of WOX1 and the Tyr33 phosphorylation in WOX1 play an important role in C1q-induced apoptosis.

To determine the role of p53 and WOX1 in C1q-regulated cell death, DU154 cells were transiently overexpressed with both p53 and WOX1. The DNA fragmentation significantly increased in the combination compared to p53 or WOX-1 alone expressing cells, suggesting that tumor suppressor p53 physically interacts with WOX1 to induce apoptosis. However, alterations of human *WOX* gene occur most frequently in the prostate. Therefore, prostate tissues were examined for the expression of C1q. The immunofluorescence comparison of the age-matched prostate tissue with the benign prostatic hyperplasia (BPH) and prostate cancer showed that C1q was significantly down-regulated in the later tissues. These findings raised a question whether down regulation of C1q *in vivo* may reduce the activation of tumor suppressors, which would subsequently result in better survival of the prostate cancer cells. Therefore, when DU145 cells were cultured overnight under serum free conditions, in the presence of 1% normal human serum or 1% human serum deficient in C1q, C6, C7, C8, or C9, it was noted that C1q and C6 were essential for the expression of WOX2 (isoform of WOX1) and p-ERK and remainder conditions did not support the expression of these proteins. These observations were further confirmed by immunofluorescence microscopy and western blot that showed the downregulation of p-WOX1 under C1q or C6 free conditions, and hence no apoptosis was seen. Similarly, p53 nuclear accumulation was reduced in the C1q or C6 free conditions whereas C9 free conditions promoted the accumulation of p53 in the nuclei suggesting that C9 restricts the p53 activation. C1q and C6 were also shown to block JNK1 activation, which is known to block the apoptotic function of WOX1 *in vitro*.

In addition, hyaluronic acid was shown to activate STAT3 in the DU145 cells when cultured in the C1q deficient conditions, which causes metastasis by up regulating the STAT3 phosphorylation, suppression of p53, and WOX1 activation. The induction of apoptosis by C1q in the cells overexpressing WOX1 such as breast cancer cells (MCF7), neuroblastoma SH-SY5Y, and SK-N-SH cells was also established. It appears that C1q plays a key role in the activation of tumor suppressor WOX1, which is required for blocking the cancer cell proliferation. C1q did not enhance the p53-mediated apoptosis; however, p53 and WOX1 balanced binding interaction results in their synergistic effect in inducing apoptosis in response to the C1q treatment. The downregulation of C1q enhances prostate hyperplasia due to a failure of WOX1 activation failure and increased activation of STAT3. It is worth pointing out here that the pro-apoptotic effect of C1q observed on human prostate cancer cells is an exceptional and novel phenomenon reported. C1q has often been detected at tumor site where its expression is associated with tumor progression.

## Role of C1q in Aging

**Figure 5:**
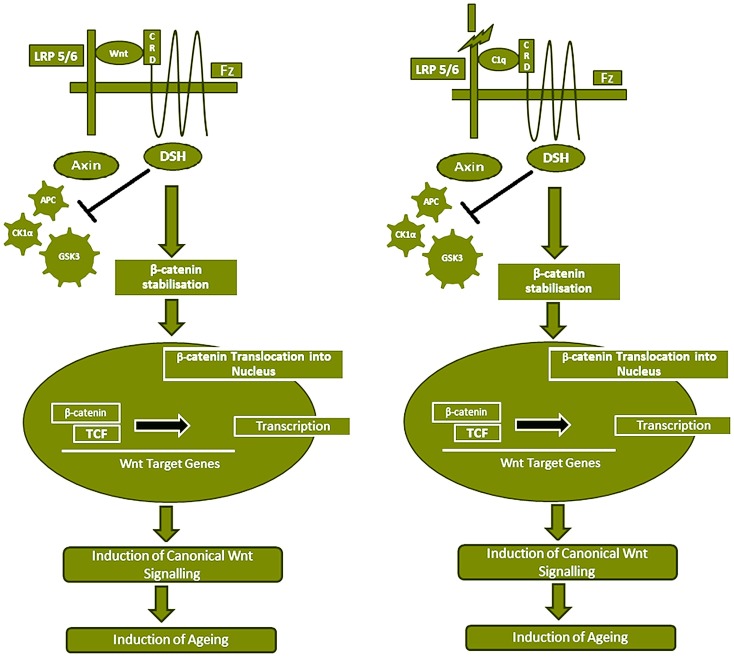
**Serum C1 competes with Wnt for Fz receptor binding, resulting in Wnt canonical pathway activation**. Wnt binds to Fz receptor and LRP5/6 which causes β-catenin destruction complex (APC, PP2A, GSK3, and CK1α) by recruiting the Axin component of destruction complex to the cytoplasmic tail of the Wnt co-receptor LRP. This results in stabilization and accumulation of β-catenin in the cytoplasm, which eventually gets translocated into the nucleus to act as a transcriptional co-activator of the TCF/LEF family and the Wnt canonical pathway is activated, which has been shown to induce aging. C1q concentration in the serum increases with age. C1q competes with 200-fold higher binding affinity with Wnt for Fz receptor and stimulates C1s-dependent cleavage of the ectodomain of LRP6. The C1q binding to Fz receptor also blocks the β-catenin destruction complex (APC, PP2A, GSK3, and CK1α) and results in stabilization of β-catenin in the cytoplasm, which is then translocated into the nucleus. Subsequently, canonical Wnt signaling is activated.

Serum C1q concentration and expression increases with aging, which causes impaired regeneration capacity of various tissues by activating canonical Wnt signaling pathway (33). Wnt signaling is known to play a key role in tissue development including stem cell self-renewal, degenerative diseases, and carcinogenesis. During canonical Wnt signaling pathway, Wnt binds to the two types of receptors, Frizzled (Fz) family of serpentine proteins and the single-transmembrane protein low-density lipoprotein receptor-related protein 5/6 (LRP5/6), which causes the disruption of the β-catenin destruction complex by recruiting the Axin component of destruction complex to the cytoplasmic tail of the Wnt co-receptor LRP. The β-catenin destruction complex includes proteins: Axin, adenomatosis poluposis coli (APC), protein phosphatase 2A (PP2A), glycogen synthase kinase 3 (GSK3), and casein kinase 1 α (CK1α). Therefore, canonical Wnt signaling pathway causes the β-catenin cytosolic accumulation and translocation into the nucleus to act as a transcriptional co-activator of the T cell factor/lymphoid enhancer factor (TCF/LEF) family (Figure 5).

C1q has been shown to activate the canonical Wnt signaling by binding to the Fz receptor and stimulating C1s-dependent cleavage of the ectodomain of LRP6. This C1q-induced activation of Wnt signaling subsequently stabilized the cytosolic β-catenin and increased the expression of Axin 2, a known target gene of canonical Wnt signaling. A heterologous competition assay demonstrated that C1q competed with Wnt for binding to Frizzled-8 (Fz8) cysteine-rich domain (CRD) with comparable affinity, however, 200-fold higher EC_50_ of C1q was observed than Wnt3A. This observation was further supported when an increase in the levels of *Axin 2* mRNA was observed in various tissues of the 2 years old wild-type mice compared with 2 months old and C1q^−/−^ mice, suggesting the relevance of C1q-induced canonical Wnt signaling activation in the aged animals. Similar trend was observed in the isolated satellite cells and fibroblasts from the muscles of young and old mice treated with C1q suggesting a decreased regenerative capacity of skeletal muscles. In addition, with increasing age, an increased amount of the cleaved product of LRP6 was detected in the serum of wild-type mice but not in C1q^−/−^ mice. These observations strongly suggest the physiological relevance of C1q-induced activation of Wnt signaling. However, the extent of this activation is dependent on various factors such as concentration of C1q, target cell exposure, expression level of Fz receptors, LRP5/6 co-receptors, C1r, C1s, and C1 inhibitor in the target cells. In conclusion, the serum C1q concentration increases with aging, which induces canonical Wnt signaling pathway and thereby C1q mediates impaired regenerative capacity of skeletal muscle in aged animals.

## Role of C1q in Wound Healing

C1q deposited on the endothelial cells of wound healing skin play a crucial role in promoting angiogenesis by inducing permeability, increased rate of proliferation and tube formation (39, 118). The presence of C1q in the endothelium and stroma of the granulated tissue and its absence in the intact skin was confirmed by immunmohistochemical analysis. C1q transcripts could be seen in stromal cells and vascular endothelium of the lesions, whereas it was undetectable in the cells of intact skin. C1q (10 μg/ml) has been shown to promote permeability, proliferation, and migration of endothelial cells. This effect of C1q appears to be mediated via its gC1q domain.

When C1q was tested using *in vitro* tube formation assay that involves growing endothelial cells and staining them for actin to visualize tube formation, C1q, like VEGF, induced marked changes in the cell pattern, with the formation of tubules assembled by elongation and joining of endothelial cells (200, 201). An *ex vivo* model of rat aortic ring assay was used to analyze the effect of C1q on vessel sprouting from aortic rings. The microvessels formation induced by C1q was visible after 6 days (more apparent after 9 days).

In a mouse model of wound healing using C1q^−/−^ mice, the animals were sacrificed after 14 days post-surgery and skin samples were collected to analyze for the presence of blood vessels. A limited number of small vessels were observed in the wound healing of the C1q^−/−^ mice in comparison to the normal angiogenesis observed in wild-type control. Local application of C1q to the wound of C1q^−/−^ restored vessel formation to the level found in WT mice (39). It appears that this angiogenic property of C1q is complement-independent.

## Perspectives

C1q plays an important role in the clearance of pathogens. C1q also binds to Gram-positive (*S. pyogenes*) and Gram-negative (*E. coli*) (Table 1) and facilitates clearance of bacterial cells (34). However, it can also be used as an escape mechanism by pneumococci, which enhances adherence and invasion in both epithelial and endothelial cells (Table 1). Although the binding to the pathogen is via surface-exposed protein(s) and the gC1q domain, the adherence and invasion is facilitated by the interaction of C1q collagen region with the host cell-surface receptor, implying that C1q acts as a link between the host and pneumococci (34). Pneumococcal endopeptidase O (PepO) binds strongly to C1q causing activation of classical pathway and consuming the complement components, which allows escape mechanism for Gram-positive species *Streptococcus pneumoniae*. PepO also binds to the C4BP, an inhibitor of the classical pathway (37).

C1q has long been considered a prototypical innate immune molecule with a range of diverse ligands and functions. C1q and its pathophysiological importance can never be over-estimated. C1q involvement in apoptotic cell clearance and its deficiency linked to the development of lupus have been some of the path breaking observations. Its neuroprotective role in clearing protein aggregates in the CNS and perhaps exaggerating neuroinflammation is firmly established. The local synthesis of C1q by immune and non-immune cells and its functions independent of other complement proteins have put the molecule at the forefront of the homeostatic machinery. Its interaction with novel receptors linked with apoptosis induction and aging are exciting areas for further investigation. The roles of C1q in the CNS pathophysiology and development are some of the highlights of complement research in last decade.

## Conflict of Interest Statement

The authors declare that the research was conducted in the absence of any commercial or financial relationships that could be construed as a potential conflict of interest.

## Abbreviations

CERT, ceramide transporter protein; CJD, Creutzfeldt–Jakob disease; CNS, central nervous system; CRP, C-reactive protein; DAF, decay-accelerating factor; DC, dendritic cells; dsDNA, double-stranded DNA; FDC, follicular dendritic cells; GABA, gamma-aminobutyric acid; HIV, human immunodeficiency virus; IL-6, interleukin-6; HTLV-1, human T-lymphotropic virus 1; LAIR, leukocyte-associated Ig-like receptor; LPS, lipopolysaccharides; MAC, membrane attack complex; MCP, membrane co-factor protein; NGF, nerve growth factor; pDC, plasmacytoid dendritic cell; PrP, prion protein; PrPSc, scrapie-associated prion protein; PS, phosphatidylserine; RGC, retinal ganglionic cells; ROS, reactive oxygen species; SLE, systemic lupus erythematosus; TNF-α, tumor necrosis factor-alpha; TGF-β, transforming growth factor-beta.
